# Adverse neonatal outcomes and associated factors among mothers who gave birth through induced and spontaneous labor in public hospitals of Awi zone, Northwest Ethiopia: a comparative cross-sectional study

**DOI:** 10.1186/s12884-023-05631-4

**Published:** 2023-05-02

**Authors:** Melaku Laikemariam, Almaz Aklilu, Fikadu Waltengus, Melkamu Addis, Wubishet Gezimu, Fekadu Baye, Temesgen Getaneh

**Affiliations:** 1 Department of Midwifery, College of Health Science, Debre Markos University, Debre Markos, Ethiopia; 2 Department of Midwifery, School of Health Sciences, College of Medicine and Health Science, Bahr Dar University, Bahr Dar, Ethiopia; 3 Department of Nursing, College of Health Science, Mettu University, Mettu, Ethiopia

**Keywords:** Adverse neonatal outcomes, Induced labor, Spontaneous labor, Ethiopia

## Abstract

**Background:**

Adverse neonatal outcomes are one of the most common causes of neonatal mortality and morbidity. Empirical evidence across the world shows that induction of labor potentiates adverse neonatal outcomes. In Ethiopia, there has been limited data that compares the frequency of adverse neonatal outcomes between induced and spontaneous labor.

**Objectives:**

To compare the prevalence of adverse neonatal outcomes between induced and spontaneous labor and to determine associated factors among women who gave birth in public hospitals of Awi Zone, Northwest Ethiopia.

**Methods:**

A comparative cross-sectional study was conducted at Awi Zone public hospitals from May 1 to June 30, 2022. A simple random sampling technique was employed to select 788 (260 induced and 528 spontaneous) women. The collected data were analyzed using statistical package for social science (SPSS) software version 26. The Chi-square test and an independent t-test were used for categorical and continuous variables, respectively. A binary logistic regression was used to assess the association between the outcome and explanatory variables. In the bivariate analysis, a p-value ≤ 0.2 at a 95% confidence interval was used to consider the variables in the multivariate analysis. Finally, statistical significance was stated at a p-value of less than 0.05.

**Result:**

The adverse neonatal outcomes among women who gave birth through induced labor were 41.1%, whereas spontaneous labor was 10.3%. The odds of adverse neonatal outcomes in induced labor were nearly two times higher than in spontaneous labor (AOR = 1.89, 95% CI: 1.11–3.22). No education (AOR = 2.00, 95% CI: 1.56, 6.44), chronic disease (AOR = 3.99, 95% CI: 1.87, 8.52), male involvement (AOR = 2.23, 95% CI: 1.23, 4.06), preterm birth (AOR = 9.83, 95% CI: 8.74, 76.37), operative delivery (AOR = 8.60, 95% CI: 4.63, 15.90), cesarean section (AOR = 4.17, 95% CI: 1.94, 8.95), and labor complications (AOR = 5.16, 95% CI: 2.90, 9.18) were significantly associated factors with adverse neonatal outcomes.

**Conclusion and recommendation:**

Adverse neonatal outcomes in the study area were higher. Composite adverse neonatal outcomes were significantly higher in induced labor compared to spontaneous labor. Therefore, it is important to anticipate the possible adverse neonatal outcomes and plan management strategies while conducting every labor induction.

## Introduction

By its nature, childbirth is a risky event for both the mother and the fetus, regardless of the mode of delivery [1]. In the natural (spontaneous) onset and termination of labor, there is very little chance of complications. However, technological labor interventions, including induction, carry considerable risk. Induction of labor is the artificial initiation of uterine contractions to accomplish delivery prior to the commencement of spontaneous labor [1–3]. Induction of labor is not risk-free, and many women find it uncomfortable. There are several ways to perform the induction procedure, including surgical, medical, and combined methods [4]. Each of these procedures entails possible hazards for the mother and the newborn [5].

While being a contentious obstetric procedure, induction of labor undoubtedly reduces some risks associated with an ongoing pregnancy, such as intrauterine fetal death (IUFD) for an unknown reason if carried out electively [6]. According to the World Health Organization (WHO) survey, about 9.6% of the deliveries involved labor induction. The proportion is higher in technologically advanced countries. In developed countries, induction of labor currently accounts for around one-quarter of all deliveries at term [3].

In comparison to watchful follow-up, induction of labor is associated with increased perinatal mortality, neonatal hospitalizations, and low Apgar scores. The perinatal mortality rate encompasses both stillbirths (fetal death in the intrapartum period) and early neonatal deaths [7]. Adverse neonatal outcomes occur far more frequently in induced labor [8]. Two studies, retrospective cohort and meta-analysis, conducted in Australia, identified induction of labor with an increased risk of adverse neonatal outcomes [9, 10]. Another retrospective cohort study carried out in the United Kingdom (UK) revealed that the rate of neonatal admission to neonatal intensive care was elevated when labor was induced [11]. A prospective cohort research carried out in Nigeria indicated that induced labor was associated with a greater rate of neonatal intensive care unit (NICU) admission [12]. A cross-sectional study carried out in Ethiopia’s Tigray region revealed a statistically significant association between poor delivery outcomes and labor induction [13].

The adverse outcomes in induced and spontaneous labor were significantly associated with different socio-demographic, obstetric, and individual factors. Adverse neonatal outcomes are significantly associated with maternal education, age, maternal race, antenatal care (ANC), parity, gestational, income, birth weight, cervical status, pregnancy-induced hypertension, and pre-existing chronic disease, according to empirical evidence from around the world [14–22].

Adverse neonatal outcomes are a major public health problem and far more frequent following induction of labor [14, 23], and they are the major causes of neonatal morbidity and mortality [24]. In Ethiopia, the adverse neonatal outcome was found to be significantly high [15, 25]. As in any of other low-resource country, adverse neonatal outcomes are a major public health problem in both induced and spontaneous labor in Ethiopia [14].

Ethiopia has introduced a variety of strategies and has made a strong effort to improve neonatal health (to reduce neonatal mortality and morbidity), however, the country still has high neonatal mortality and morbidity, which is substantially higher than the the sustainable development goal’s (SDG’s) target by 2030 [26]. Robust and up-to-date evidence is compulsory to strengthen the continuing efforts to reduce adverse neonatal outcomes, the major cause of morbidity and mortality.

Even though there have numerous studies been carried out on adverse newborn outcomes in Ethiopia, most of these studies did not compare the prevalence of those outcomes in induced and spontaneous labor. The current study therefore aimed to compare the prevalence of adverse neonatal outcomes and determine associated factors among mothers who gave birth through induced and spontaneous labor in public hospitals of the Awi Zone, Northwest Ethiopia. Our study’s main hypotheses were that there is a difference between spontaneous and induced labor in adverse neonatal outcomes.

## Method and materials

### Study design, study period, and study setting

A comparative cross-sectional study was conducted from May 1 to June 30, 2022, in public hospitals in the Awi Zone. The Awi zone is located in the Amhara Regional State, northwest of Ethiopia. The capital of the zone is Injibara town, located 114 and 445.9 km from Bahr Dar city, the capital of Amhara Regional State, and Addis Ababa, the capital of Ethiopia, respectively. In the zone, there are five public hospitals and forty-seven health centers that serve a total population of around 1,077,144 [27]. The annual delivery report from the zone’s public hospitals was 10,547 the year before this survey.

### Population

All postpartum mothers who gave birth in the public hospitals of Awi Zone during the survey period were considered the source population, whereas systematically selected mothers from similar settings were considered the study population.

### Eligibility criteria

All selected postpartum mothers who agreed to participate were included in the study. However, mothers who suffered from intrauterine fetal death or were critically ill during the survey were excluded from this study. In addition, mothers who were referred to another hospital while being interviewed were excluded.

### Sample size determination

The sample size was calculated assuming 50% for P1 and P2, a 5% degree of freedom, and a 95% two-sided level of confidence as follows:

**Table:** 

Anticipated population proportions	P1 and P2
Confidence level	100(1-a)%
Absolute precision required on either side of the true value of the difference between P1 and P2 100(1-a)% the proportions (in percentage points)	D
Intermediate value	V = P1 (1-Pd + P2 (1-P2)

$$N={(Z1}^{2}-@/2\left) \right[\text{P}1 (1- \text{P}1) + \text{P}2(1- \text{P}2)]/\text{D}2$$

$$N={(Z1}^{2}-@/2) V/\text{D}2$$

Where, V = P1 (1-P1) + P2 (1-P2), Intermediate value.

$$\begin{array}{l}N = {\left( {1.96} \right)^2}\,[0.5(1 - 0.5) + 0.5(1 - 0.5)]/{\left( {0.05} \right)^2}\\{\mkern 1mu} {\mkern 1mu} = 806.72 \sim 807\end{array}$$

Based on the above calculation, the total sample size for this study was 807, considering a 5% non-response rate (538 spontaneous and 269 induced women with a 2:1 ratio).

### Sampling procedure

**Fig. 1:**
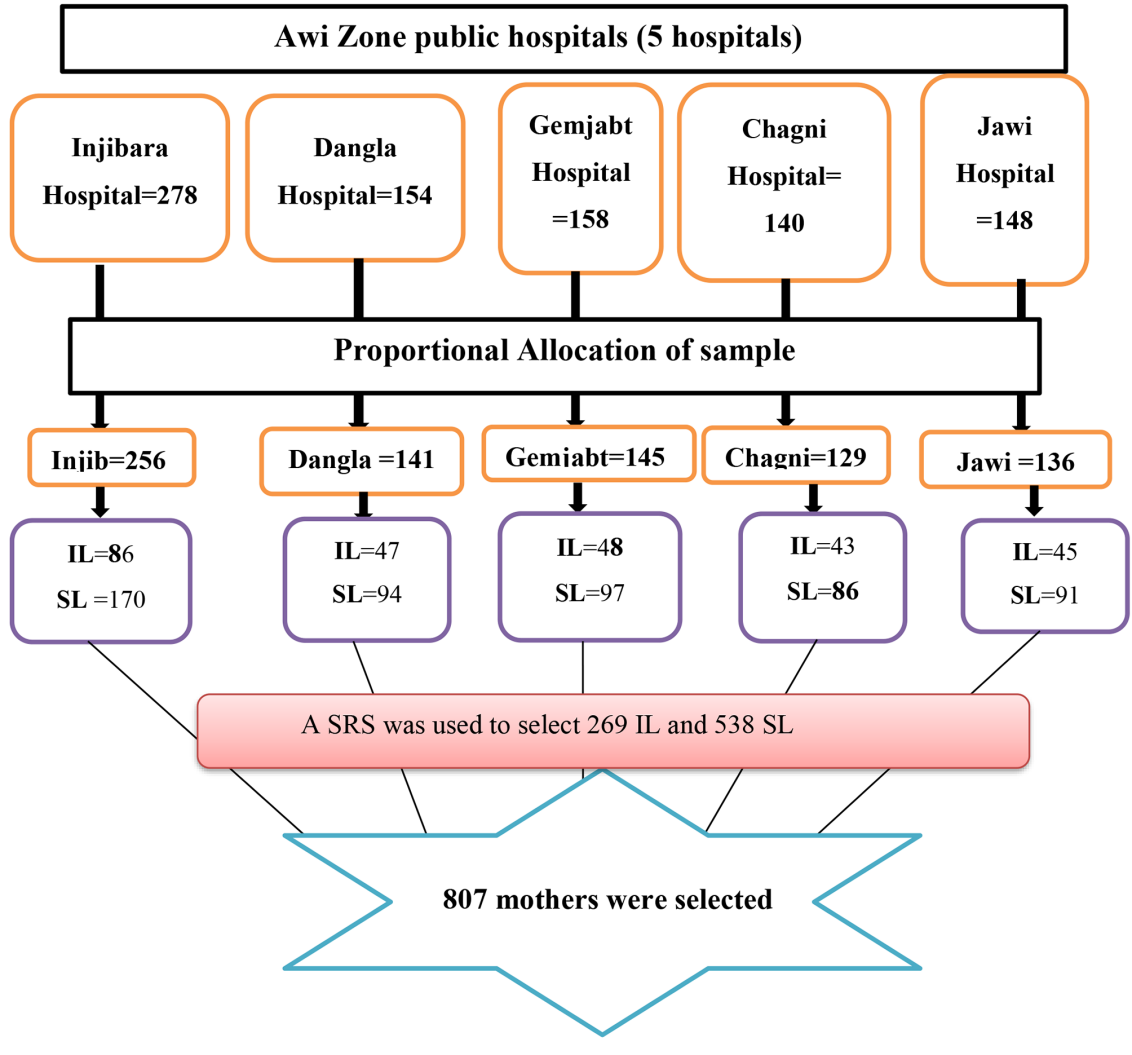
Schematic presentation of sampling procedure to select women from public hospitals in the Awi Zone, Ethiopia 2022 **Note:** **IL:** Induced labor. **SL:** Spontaneous labor. **SRS:** Systematic random sampling.

All five public hospitals found in the Awi Zone were included in the study. The sample was proportionally allocated to each hospital based on the previous year’s two-month average number of delivery reports for the respective hospitals. Then a systematic random sampling technique was used to select participants from each public hospital. The Kth value for each hospital was calculated by dividing the two-month (May and June) average delivery of the previous year preceding this survey by the total sample size, which is ~ 2 for all hospitals (Fig. 1). The first mother was selected separately for each hospital using a simple random sampling technique among mothers who gave birth on the first day of the postpartum period.

### Study variables

The comparison of the prevalence of adverse neonatal outcomes in induced and spontaneous labor was the outcome variable of this study, whereas socio-demographic characteristics (age, residence, educational status, occupation, religion, ethnicity, marital status, and monthly income), reproductive and obstetric characteristics (gravidity, parity, gestational age, ANC, Iron Folate supplementation, bad obstetric history, method of induction, pregnancy complications, MUAC, and hemoglobin level, male involvement in health-seeking behavior) and behavioral and medical-related characteristics (prenatal substance use history, existence of chronic disease, and history of malaria) were the explanatory variables tested in this study.

### Operational and term definitions

Adverse (unfavorable) neonatal outcome: was referred as the occurrence of at least one of the following: need of resuscitation following delivery, low Apgar score at first or fifth minutes, fetal death during intrapartum, immediate neonatal death, RDS, birth asphyxia, NICU admission, and neonatal jaundice within 24 h of delivery [16, 28–33].

#### Stillbirth

was referred as the death of a fetus before delivery, but after initiation of labor (fetal death during the intrapartum time before delivery) [34].

#### Immediate (early) neonatal death

the death of the newborn or death of the neonate within the first 24 h of life [35].

**Bad obstetric history**: was considered when a woman had at least one of the following conditions in a previous pregnancy: still birth, early neonatal death, and recurrent abortion [36].

#### Favorable cervical condition

when the Bishop score is ≥ 6, cervical condition and induction is likely to succeed [2].

**Unfavorable cervical condition:-** when the Bishop score is ≤ 5, cervical status is unlikely to yield for induction [4].

### Data collection tools and technique

Ten data collectors (two professionals for each hospital) from the governmental health institutions (five midwives and five health extension workers) collected the data using a pretested structured questionnaire and checklist that was prepared in Epicollect-5 software. The questionnaire was adapted from similar previous studies where its validity was tested [37]. The data collectors approached the study participants at the time of exit or after 24 h of the postpartum period and conducted face-to-face interviews.

### Data quality assurance

Two weeks preceding the actual study, a pretest was conducted among 5% (19 spontaneous and 9 induced labor cases) of the total sampled population in Durbtie primary hospital. Based on the pretest data, necessary amendments in the sequence and wording of the questionnaires were made. Confusing and unclear questions were checked and edited accordingly before actual data collection. Regular checkups for completeness and consistency of the data were performed on a daily basis. To ensure a clear understanding for the participants and to obtain correct responses, the English version of the questionnaire was translated to Amharic (a local language) and then retranslated back to English by two language experts. The two-day training was given to the data collectors and supervisors. After the data were collected, they were downloaded into excel in CSV format, then exported to SPSS for analysis. In addition, during data analysis, the data were cleaned carefully and any missing values were handled carefully.

### Data processing and analysis

Data were cleaned to ensure completeness, consistency, the absence of missing values, and appropriate variable coding. The adverse neonatal outcomes and socio-demographic characteristics were analyzed through descriptive chi-square cross-tabulation analysis using statistical package for social science (SPSS) software version 26. The Chi square test and an independent t-test were used to compare categorical and continuous variables between induced women and spontaneously delivered women, respectively. In addition, a binary logistic regression analysis was performed to test the associations. In the bivariate analysis, a p-value ≤ 0.2 at 95% CI was used. A significant association was stated when a p-value was less than 0.05 at a 95% CI in multivariate analysis after controlling for potential cofounders. A model fit test was conducted using the Hosmer and Lemeshow test, and multicollinarity diagnostics was conducted through linear regression with backward stepwise conditional analysis using variance inflation factor (VIF). Data presentation techniques such as percentages, frequency distribution tables, and figures were used.

## Results

### Socio-demographic characteristics of participants

From the total of 807 sampled populations, 788 (528 spontaneous and 260 induced women) participants were involved in the study, giving a response rate of 97.6%. The mean age participants who experienced induced labor was 27.91 (SD = ± 6.12), whereas the mean age of participants who gave birth through spontaneous labor was 26.92 (SD = ± 5.47). About 145 (61.4%)) of participants who gave birth through spontaneous labor had no formal education. In women whose labor induced, 214(82.3%) women had male partner involvement in their health seeking behavior compared to 474(89.7%) women in spontaneous labor (Table 1).

**Table 1:** Socio-demographic characteristics of mothers who gave birth through induced and spontaneous labor in public hospitals of Awi Zone, Northwest Ethiopia, 2022

Variables		Induced labor (n = 260)		Spontaneous labor (n = 528)	Total (n = 788)	
		Frequency (%)		Frequency (%)	Frequency (%)	**χ**^**2**^,Asymptotic Significance (2-sided)
Age of respondent’s	17–19 years(< 20 years)	21(31.3%)		46(68.7%)	67(8.5%)	0.032
	20–34 years	196(31.4%)		429(68.6%)	625(79.3%)	
	>= 35 years	43(44.8%)		53(55.2%)	96(12.2%)	
Residence	Rural	106(33%)		215(67%)	321(40.7%)	0.989
	Town	154(33%)		313(67%)	467(59.3%)	
Marital status	Single	5(29.4%)		12(70.6%)	17(2.2%)	< 0.001
	Married /union	229(31.2%)		506 (68.8%)	735(93.3%)	
	Others^a^	26(72.2%)		10(27.8%)	36(4.6%)	
Maternal education	No education	91(38.6%)		145(61.4%)	236(29.9%)	0.173
	Primary education	63(30%)		147 (70%)	210(26.6%)	
	Secondary education	61(30%)		142(70%)	203(25.8%)	
	> Secondary education	45(32.4%)		94(67.6%)	139(17.6%)	
Male involvement	No	44(44%)	56(56%)		100(12.7%)	0.006
	Yes	214(31.1%)	474(68.9%)		688(87.3)	
Religion	Orthodox	219 (32.2%)		461(67.8%)	680(86.3%)	0.489
	Muslims	33(38.4%)		53(61.6%)	86(10.9%)	
	Others^b^	8 (36.4%)		14(63.6%)	22(2.8%)	
Ethnicity	Awi/Agew	133(31.4%)		291(68.6%)	424(53.8%)	< 0.001
	Amhara	88 (29.6%)		209(70.4%)	297(37.7%)	
	Others^c^	39 (58.2%)		28(41.8%)	67(8.5%)	
Maternal occupation	House wife	70 (33.3%)		140(66.7%)	210(26.6%)	0.977
	Farmer	83(32.7%)		171(67.3%)	254(32.2%)	
	Governmental employee	31(34.8%)		58 (65.2%)	89(11.3%)	
	Others^d^	76(32.3%)		159(67.7%)	235(29.9%)	
Family monthly income(ETB)	< 500 ETB	77(41.4%)		109(58.6%)	186(23.6%)	0.039
	500–1000 ETB	92(29.3%)		222(70.7%)	314(39.8%)	
	1001–2000 ETB	26(33.8%)		51(66.2%)	77(9.8%)	
	> 2000 ETB	65(30.8%)		146(69.2%)	211(26.8%)	

Note: ^a^ Divorced and widowed, ^b^Oromo and Benshagule Gumez, ^c^Muslim and no religion, ^d^Student, merchant and daily laborers/private employee, the minimum age of respondent’s used in the study was > 17 years

### Behavioral and pre-existing medical problems

Of the participants, 54(20.7%) induced women had prenatal substance use compared to 66(12.5%) women who gave birth through spontaneous labor. The percentage of chronic disease in induced and spontaneous labor was 28(10.8%) and 26 (4.9%), respectively (Table 2).

**Table 2:** Lifestyle and medical related characteristics of mothers who gave birth through induced and spontaneous labor in public hospitals of Awi Zone, Northwest Ethiopia, 2022

Variables		Induced labor (n = 260)	Spontaneous labor (n = 528)	Total (n = 788)	
		Frequency (%)	Frequency (%)	Frequency (%)	**χ**^**2**^,Asymptotic Significance (2-sided)
Prenatal substance use	Yes	54(45.8%)	64(54.2%)	118 (15%)	0.001
	No	206(30.7%)	464(69.3%)	670 (85%)	
Chronic disease	Yes	28(51.9%)	26(48.1%)	54(6.9%)	< 0.001
	No	232(31.6%)	502(68.4%)	734(93.1%)	
Types of pre-existing chronic disease^R^	Pre-gestational Diabetes mellitus	7(87.5%)	1(12.5%)	8 (1%)	< 0.001
	Chronic hypertension	8 (80%)	2(20%)	10(1.3%)	
	Anemia	3(50%)	3(50%)	6(0.8%)	
	Others^*^	10(33.3%)	20(66.7%)	30(3.8%)	
History of malarial infection	Yes	79(37.3%)	133(62.7%)	212(26.9%)	0.122
	No	181(31.4%)	395(68.6%)	576(73.1%)	

Note: ^*^Asthma, tuberculosis and HIV/AIDS, ^**R**^More than one choice possible

### Obstetric characteristics

The proportion of bad obstetric history among women who gave birth through induced and spontaneous onset of labor was 54(20.7%) and 76 (14.4%), respectively. Two hundred four (78.5%) induced women were supplied with iron with folic acid compared to four hundred forty-eight (84.8%) spontaneously labored study participants. About 520 (98.5%) spontaneously labored women had ANC follow-up. The mean GA of induced women and spontaneously delivered mothers was 39.61(SD = ± 2.06) and 38.04(SD = ± 1.64), respectively. Among women whose labor was induced, 75 (28.8%) women encountered complications during labor delivery, but only 41(7.8%) spontaneously delivered women faced complications during labor delivery (Table 3).

**Table 3:** Obstetrics characteristics of mothers who gave birth through induced and spontaneous labor in public hospitals of Awi Zone, Northwest Ethiopia, 2022

Variables		Induced labor (n = 260)	Spontaneous labor (n = 528)	Total (n = 788)	
		Frequency (%)	Frequency (%)	Frequency (%)	χ^2^, Asymptotic Significance (2-sided)
Gravidity	Primigravida	104(32.7%)	214 (67.3%)	318(40.4%)	0.556
	Multigravida	124(32.2%)	261(67.8%)	385(48.9%)	
	Grand multigravida	32(37.6%)	53(62.4%)	85(10.8%)	
Parity	Primipara	104(32.5%)	214(67.3%)	318(40.4%)	0.727
	Multipara	125(32.5%)	260(67.5%)	385(47.5%)	
	Grand multipara	31(36.5%)	54(63.5%)	85(10.8%)	
Bad obstetric history	Yes	54(41.5%)	76(58.5%)	130(16.5%)	0.023
	No	206(31.3%)	452(68.7%)	658 (83.5%)	
Types of bad obstetric history^R^	Abortion	40(45.5%)	48(54.6%)	88(67.7%)	0.058
	Immediate neonatal death	5(27.8%)	13(72.2%)	18(13.8%)	
	Stillbirth and IUFD	9(37.5%)	15(62.5%)	24(18.5%)	
ANC follow up	No	6(42.9%)	8(57.1%)	14(1.8%)	0.428
	Yes	254(32.8%)	520(67.2%)	774(98.2%)	
GA of ANC initiation	After 12th weeks	182(30.1%)	423(69.9%)	605(78.2%)	0.002
	Within 12th weeks	72(42.6%)	97(57.4%)	169(21.8%)	
Number of ANC visit	1–3 ANC visit	74(40.2%)	110(59.8%)	184(23.8%)	0.014
	>=4 ANC visit	180(30.5%)	410(69.5%)	590(76.2%)	
TT vaccination	No	22(34.9%)	41(65.1%)	63(8%)	0.735
	Yes	238(32.8%)	487(67.2%)	725(92%)	
Iron with folic acid supplementation	No	56(41.2%)	80(58.8%)	136(17.3%)	0.026
	Yes	204(31.3%)	448 (68.7%)	652(82.7%)	
Duration of iron with folic acid supplementation	< 3 months	74(28%)	190(72%)	264(40.5%)	0.025
	>= three months	130(33.5%)	258(66.5%)	388(59.5%)	
Pregnancy complications	Yes	141(74.2%)	49(25.8%)	190(24.1%)	< 0.001
	No	119(19.9%)	479(80.1%)	598 (75.9%)	
Types of pregnancy complications^R^	Pregnancy Induced hypertension	84(82.4%)	18(17.6%)	102 (53.7%)	< 0.001
	Antepartum hemorrhage(APH)	26(60.5%)	17(39.5%)	43(22.6%)	
	PROM	12(60%)	8(40%)	20 (10.5%)	
	Others^*^	19(76%)	6(24%)	25(13.2%)	
Maternal MUAC	=<22 cm	54(32.1%)	114(67.9%)	168(21.3%)	0.791
	>=23 cm	206(33.2%)	414(66.8%)	620(78.7%)	
Maternal Hgb	=<10 mg/dl	15(57.7%)	11(42.3%)	26(3.3%)	0.006
	>=11 mg/dl	245(32.2%)	517(67.8%)	762(96.7%)	
Gestational age	Preterm	13(54.2%)	11(45.8%)	24(3%)	< 0.001
	Term	221(30%)	515(70%)	736(93.4%)	
	Post term	26(92.9%)	2(7.1%)	28(3.6%)	
MSAF	Yes	158(87.3%)	23(12.7%)	181(23%)	< 0.001
	No	102(16.8%)	505(83.2%)	607(77%)	
Mode of delivery	Instrumental delivery	84(77.1%)	25(22.9%)	109(13.8%)	< 0.001
	Emergency CS delivery	66(85.7%)	11(14.3%)	77(9. 8%)	
	SVD	110(18.3%)	492(81.7%)	602(76.4%)	
Complication during labor –delivery	Yes	75(64.7%)	41(35.3%)	116(14.7%)	< 0.001
	No	185(27.5%)	487(72.5%)	672(85.3%)	
Types of labor delivery complications^R^	Precipitated labor	42(87.5%)	6(12.5%)	48(41.4%)	< 0.001
	Prolonged labor	22(44%)	28(56%)	50(43.2%)	
	Postpartum hemorrhage	11(57.9%)	8(42.1%)	19(16.4%)	

Note: ^*^ Gestational DM and related complications, ^**R**^More than one choice possible, PROM (premature rupture of membrane), SD = standard deviation

#### Newborn characteristics

The proportion of fetal deaths in the intrapartum period and neonatal deaths in the first 24 h of birth among women who gave birth through induced and spontaneous labor was 13(5%) and 6(1.14%) deaths, respectively. The mean first-minute Apgar score among induced and spontaneously delivered newborns was 6.86 (SD = ± 1.36) and 7.44(SD = ± 0.91), respectively. Likewise, the mean fifth minute Apgar score among induced and spontaneously delivered newborns was 8.44(SD = ± 1.75) and 8.88(SD = ± 1.03), respectively, through independent T-test. The mean newborn birth weight in grams among induced and spontaneously delivered newborns was 3073.08 (SD = ± 372.78) and 3067.8 (SD = ± 323.27) grams, respectively. The significant proportion of newborns born through induced labor had a low first-minute Apgar score of 77 (29.6%) compared to the newborns delivered through spontaneous labor of 33 (6.3%). A significant percentage of newborns delivered through induced labor were admitted to the NICU compared to newborns delivered through spontaneous labor [40(15.4%) and 30 (5.7%), respectively] (Table 4).

**Table 4:** Newborn characteristics of mothers who gave birth through induced and spontaneous labor in public hospitals of Awi Zone, Northwest Ethiopia, 2022

Variables		Induced labor (n = 260)	Spontaneous labor (n = 528)	Total (n = 788)	
		Frequency (%)	Frequency (%)	Frequency (%)	**χ**^**2**^, Asymptotic Significance (2-sided)
Birth outcome	Dead	13(68.4%)	6(31.6%)	19(2.4%)	0.001
	Alive	247(32.1%)	522(67.9%)	769(97.6%)	
Sex	Male	129(36.9%)	221(63.1%)	350(44.4%)	0.039
	Female	131(29.9%)	307(70.1%)	438(55.6%)	
Newborn birth weight in gram	< 2500	12(66.7%)	6(33.3%)	18(2.3%)	0.002
	2500–4000	244 (31.9%)	520(68.1%)	764(97%)	
	> 4000	4(66.7%)	2(33.3%)	6(0.7%)	
First minute APGAR score	Low APGAR score (< 7)	77(70%)	33(30%)	110(14%)	< 0.001
	Normal APGAR score ( > = 7)	183(27%)	495(73%)	678(86%)	
Fifth minute APGAR score	Low APGAR score (< 7)	21(67.7%)	10(32.3%)	31(3.9%)	< 0.001
	Normal APGAR score ( > = 7)	239(31.6%)	518(68.4%)	757(96.1%)	
Need of resuscitation	Yes	93(69.4%)	41(30.6%)	134(17%)	< 0.001
	No	167(25.5%)	487(74.5%)	654(83%)	
NICU admission	Yes	40(57.1%)	30(42.9%)	70(8.9%)	< 0.001
	No	220(30.6%)	498 (69.4%)	718(91.1%)	
Indication of NICU admission^R^	Asphyxia	16(64%)	9(36%)	25(35.7%)	< 0.001
	Prematurity	5(41.7%)	7(58.3%)	12(17.1%)	
	Jaundice	7(50%)	7(50%)	14(20%)	
	Others^*^	12(63.2%)	7(36.8%)	19(27.2%)	
Newborn Jaundice	Yes	13(56.5%)	10(43.5%)	23(2.9%)	0.015
	No	247(32.3%)	518(67.7%)	765(97.1%)	
Newborn outcome	Favorable	153(24.4%)	474((75.6%)	627(79.6%)	< 0.001
	Unfavorable	107(66.5%)	54(33.5%)	161(20.4%)	

Note: ^*^ Infection, hypothermia and respiratory distress syndrome, ^**R**^More than one choice possible, SD = standard deviation

### Adverse neonatal outcomes

**Fig. 2:**
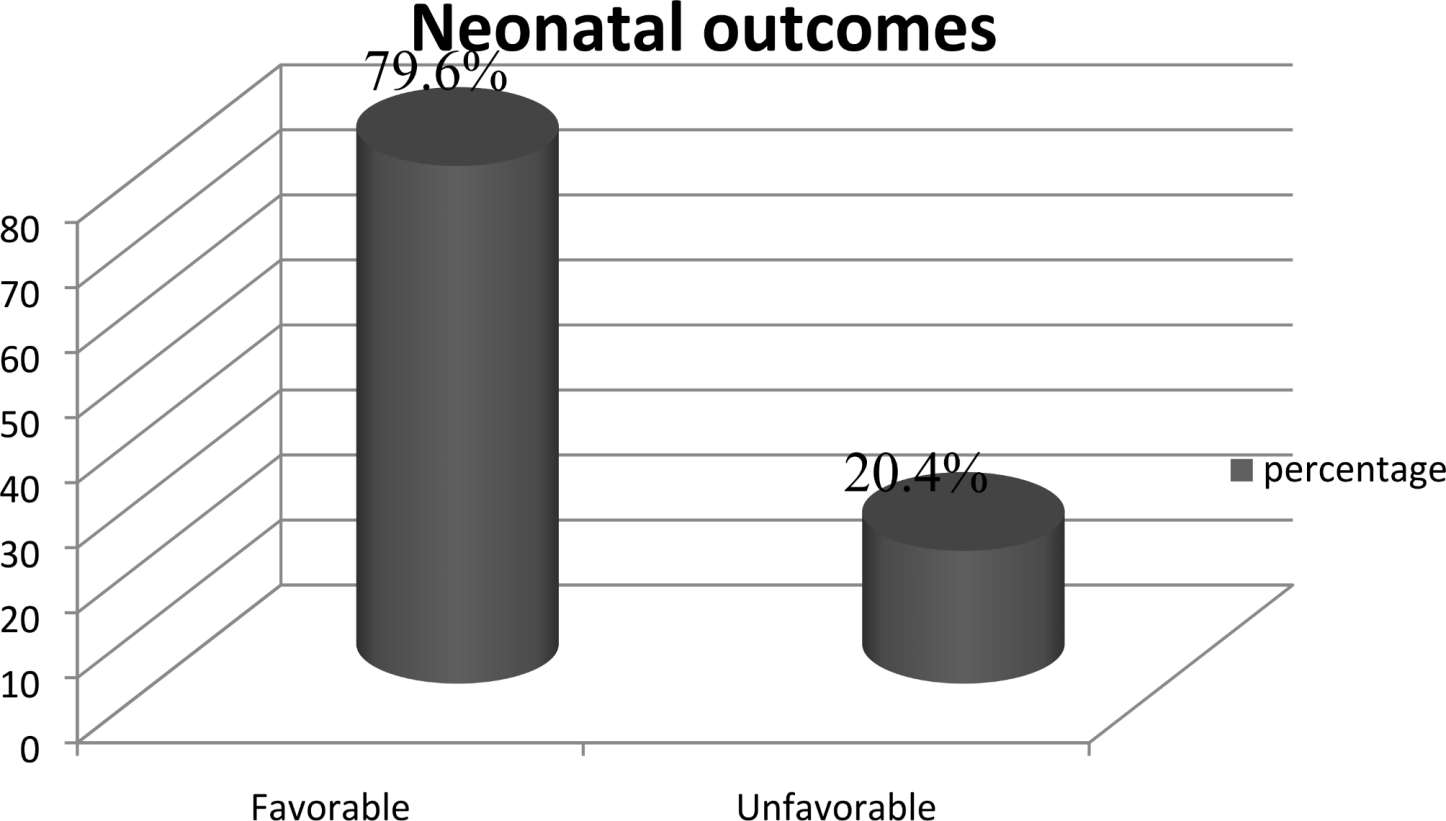
General neonatal outcomes among induced and spontaneously delivered mothers at Awi zone public hospitals, Northwest Ethiopia: 2022

**Fig. 3:**
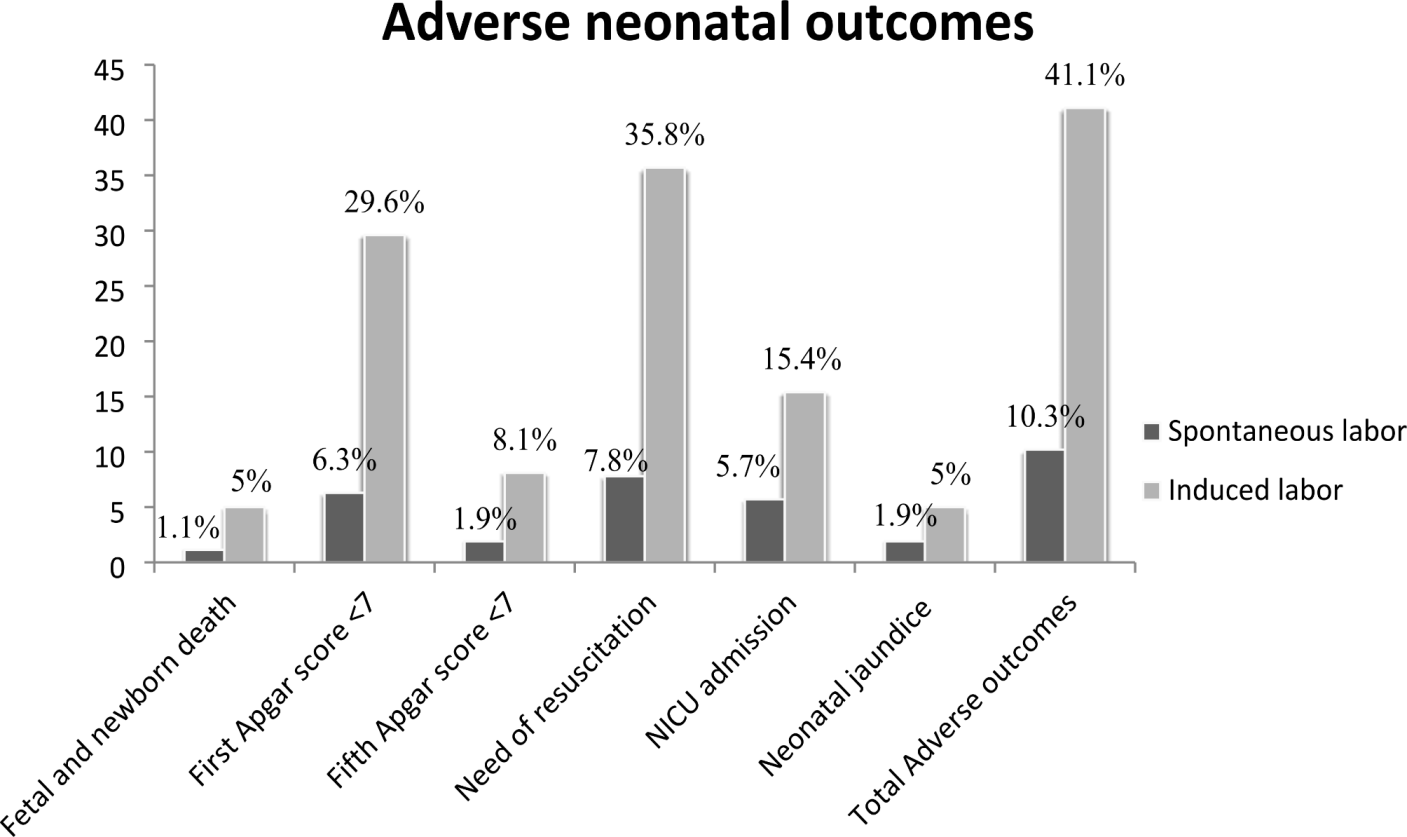
Adverse neonatal outcomes among induced and spontaneously delivered mothers at Awi zone public hospitals, Northwest Ethiopia: 2022

The adverse neonatal outcomes among women who gave birth through induction were 41.1 (95% CI: 34.8, 46.7), compared to 10.3 (95% CI: 8.1, 13.3) in women who gave birth spontaneously. The overall magnitude of adverse neonatal outcomes among women who gave birth at the public hospitals of the Awi zone was 20.4 (95% CI: 17.8, 23.0) (Fig. 2 and Fig. 3).

### Factors associated with adverse neonatal outcomes

Maternal age, marital status, educational status, monthly income, prenatal substance use, chronic disease, history of malarial infection, gravidity, parity, ANC follow up, bad obstetrical history, iron folate supplementation, complications during recent pregnancy, male involvement, hemoglobin level, gestational age, onset of labor, MSAF, mode of delivery, and complications during labor-delivery were variables that showed association with adverse neonatal outcome in the bivariate analysis at p-value ≤ 0.2.

However, in multivariable regression (after adjusting possible confounding variables), educational status, chronic disease, male involvement, gestational age, onset of labor, mode of delivery, and complications during labor-delivery were significantly associated with adverse neonatal outcomes at p-value < 0.05. Accordingly, the odds of adverse neonatal outcomes in induced labor were nearly two-fold higher than in spontaneous labor (AOR = 1.89, 95% CI: 1.11, 3.22). The likelihood of developing adverse neonatal outcomes among women who had no formal education was two times higher compared to those who had at least a secondary education (AOR = 2.00, 95% CI: 1.56, 6.44). Mothers who had chronic disease were about four times more likely to have adverse neonatal outcomes compared to mothers who had no chronic disease (AOR = 3.99, 95% CI: 1.87, 8.52). Mothers whose partners were not involved in their health-seeking behavior were around two times more likely to have adverse neonatal outcomes compared to mothers whose partners were involved (AOR = 2.23, 95% CI: 1.23, 4.06). The likelihood of developing adverse neonatal outcomes among women who encounter complications during labor-delivery was nearly five times higher compared to women who had no labor-delivery complications (AOR = 5.16, 95% CI: 2.90, 9.18). The odds of adverse neonatal outcomes among women who gave birth through cesarean section were four-fold higher compared to women who gave birth through spontaneous vaginal delivery (AOR = 4.17, 95% CI: 1.94, 8.95). The odds of adverse neonatal outcomes among women who gave birth through instrumental delivery were more than eight-fold higher than in women who gave birth through spontaneous vaginal delivery (AOR = 8.60, 95% CI: 4.63, 15.90). Moreover, the probability of developing adverse neonatal outcomes was nearly four times higher in term delivery compared to preterm (AOR = 9.83, 95% CI: 8.74, 76.37) (Table 5).

**Table 5:** Logistic regression to identify factors associated with adverse neonatal outcomes among mothers who gave birth through induced and spontaneous labor in public hospitals of Awi Zone, Northwest Ethiopia, 2022

Variables		Adverse neonatal outcomes				
		Frequency (%)		COR (95% CI)	AOR (95% CI)	p-value
		Yes	No			
Onset of labor	Induced	107(41.2%)	153(58.8%)	6.14(4.22, 8.93) *	1. 89(1.11, 3.22)	0.019
	Spontaneous	54(10.2%)	474(89.8%)	1	1	
Maternal age	< 20 years	18(26.9%)	49(73.1%)	1.66(0.93, 2.97)	1.29(0.82,3.94)	> 0.1
	20–34 years	113(18.1%)	512(81.9%)	1	1	
	>=35 years	30(31.3%)	66(68.8%)	2.06(1.278, 3.319)	1.12(0.56, 2.27)	
Maternal educational status	No education	70(29.7%)	166(70.3%)	2.021(1.2, 3.402) *	2.00(1.56, 6.44)	0.001
	Primary	35(16.7%)	175(83.3%)	0.958(0.542, 1.695)	0.92(0.82, 3.76)	0.144
	Secondary	32(15.8%)	171(84.2%)	0.897(0.502, 1.601)	0.87(0.83, 3.8)	0.138
	> secondary	24(17.3%)	115(82.7%)	1	1	
Marital status	Single	6(35.3%)	11(64.7%)	2.381(0.866, 6.549)	0.45(0.10, 1.96)	> 0.3
	Married	137(18.6%)	598(81.4%)	1	1	
	Others	18(50%)	18(50%)	4.37(2.21, 8.61)	1.17(0.42, 3.21)	
Monthly income in ETB	< 500	53(28.5%)	133(71.5%)	1.94(1.20, 3.13)	1.02(0.42, 2.47)	^>0.6^
	500–1000	59(18.8%)	255(81.2%)	1.13(0.712, 1.78)	0.91(0.43,1.94)	
	1001–2000	13(16.9%)	64(83.1%)	0.99(0.49, 1.98)	0.88(0.33,2.33)	
	> 2000	36(17.1%)	175(82.9%)	1	1	
Prenatal substance use	Yes	39(33.1)	79(66.9)	2.22(1.44, 3.41)	1.20(0.65, 2.22)	^0.55^
	No	122(18.2%)	548(81.8%)	1	1	
Chronic disease	Yes	28(51.9%)	26(48.9%)	4.87(2.76, 8.57)	3.99(1. 87, 8.52)	< 0.001
	No	133(18.1%)	601(81.9%)	1	1	
Hx of malaria infection	Yes	54(25.5%)	158(74.5%)	1.50(1.03, 2.18)	0.85(0.50, 1.45)	^0.59^
	No	107(18.6%)	469(81.4%)	1	1	
Gravidity	Primigravida	63(19.8%)	255(80.2%)	1.04 (0.71,1.51)	0.48(0.11,2.14)	> 0.3
	Multigravida	74(19.2%)	311(80.8%)	1	1	
	Grandgravida	24(28.2%)	61(71.8%)	1.65(0.97,2.83) *	1.12(0.31, 3.98)	
Parity	Primipara	66(20.8%)	252(79.2%)	1.20(0.82, 1.75)	0.93(0.54, 1.60)	> 0.2
	Multipara	69(17.9%)	316(82.1%)	1	1	
	Grandpara	26(30.6%)	59(69.4%)	2.02 (1.19,3.43)	1.87(0.60, 5.84)	
ANC follow up	No	7(50%)	7(50%)	4.03(1.39,11.65)	1.96(0.49, 7.78)	0.4
	Yes	154(19.9%)	620(80.1%)	1	1	
Bad obstetric history	Yes	48(36.9%)	82(63.1%)	2.82(1.82, 4.25)	1.48(0.81, 2.72)	0.2
	No	113(17.2%)	545(82.8%)	1	1	
Iron folate supplement	No	37(27.2%)	99(72.8%)	1.59(1.04, 2.44)	0.67(0.34, 1.30)	0.23
	Yes	124(19%)	528(81%)	1	1	
Complication during Pregnancy	Yes	77(40.5%)	113(69.5%)	4.17(2.88, 6.04)	0.98(0.55, 1.74)	0.95
	No	84(14%)	514(86%)	1	1	
Male involvement	No	39(39%)	61(61%)	2.96(1.90, 4.64)	2.23 (1.23, 4.10)	0.009
	Yes	122(17.7%)	566(82.3%)	1	1	
Maternal Hgb	<= 10 mg/dl	14(53.8%)	12(46.2%)	4.88(2.21, 10.77)	0.70(0.20, 2.46)	0.58
	>=11 mg/dl	147(19.3%)	615(80.7%)	1	1	
Gestational age	Preterm	18(75%)	6(25%)	14.07(5.48,36.16)	9.83(8.74, 76.37)	< 0.001
	Term	126(17.6%)	591(82.4%)	1	1	
	Post term	17(36.2%)	30(63.8%)	2.66(1.42, 4.97)	2.43(1.23, 6.14)	0.014
MSAF	Yes	76(42%)	105(58%)	4.45(3.06, 6.46)	0.68(0.36, 1.25)	0.21
	No	85(14%)	522(86%)	1	1	
Mode of delivery	Instrumental	61(56%)	48(44%)	12.39(7.76, 19.78)	8.60(4.63,15.90)	< 0.001
	CS	44(57.1%)	33(42.9%)	13.00(7.66, 22.05)	4.17(1.94, 8.95)	< 0.001
	SVD	56(9.3%)	546(90.7%)	1	1	
Complications during labor and delivery	Yes	75(64.7%)	41(35.3%)	12.46(8.01, 19.41)	5.16 (2.89, 9.18)	< 0.001
	No	86(12.8%)	586(87.2%)	1	1	

Note: * Significant at p ≤ 0.2 bivariate regression analysis in the variable having categories

## Discussion

This study tested the hypothesis that there is no difference between spontaneous and induced labor in the prevalence of poor newborn outcomes. Accordingly, the study confirmed that adverse neonatal outcomes were significantly higher in induced labor than in spontaneous labor. In the study, the prevalence of adverse neonatal outcomes among participants who gave birth through induced labor was found to be 41.1%, compared to 10.3% in participants who gave birth through spontaneous labor. This finding is congruent with the studies conducted in the Tigray region [38], Australia [29], Sudan [30], and India [31]. This consistency is grounded in the evidence that labor induction is associated with a range of obstetrical complications [3].

The overall prevalence of adverse neonatal outcomes found to be 20.4%. This figure is comparable with the finding from a study conducted in the Tigray Region of northern Ethiopia [13]. The reason for this similarity could be that adverse neonatal outcomes are still a public health threat and efforts should be addressed.

Regarding the specific adverse neonatal outcomes, the composite proportions of neonatal deaths in the intrapartum and immediate neonatal deaths were significantly higher among women who gave birth through induced labor (5% ) compared to those who gave birth through spontaneous labor (1.1%). This finding is in agreement with studies conducted in Australia [9], Sudan [30], and Ethiopia [30]. This might be correlated to the evidence that induced labor is associated with different early neonatal complications like birth asphyxia, respiratory complications [30], and NRFHRP [16] that result in the death of neonates in the immediate neonatal period.

The study confirmed that the percentage of Apgar scores less than 7 in the first minute (29.6%) and the fifth minute (6.3%) of delivery was significantly higher among women who gave birth through induced labor compared to women who gave birth through spontaneous labor, at 8.1% and 1.9% in the first and fifth minute, respectively. These findings were comparable to studies conducted in Australia [9], India [16], Tanzania, and Nigeria [39, 40]. This could be due to diminished utero-placental blood supply during labor induction, which could cause increased NRFHRP [41, 42] and later result in a low Apgar score. The current finding is different from a study conducted in India which found that first and fifth minute Apgar scores were significantly higher in spontaneous labor [43]. The discrepancy might be related to differences in induction procedures [38].

According to the present study, more neonates born through induced labor (35.8%) required immediate resuscitation after delivery compared to those born through spontaneous labor (7.8%). This figure is comparable to the studies conducted in Switzerland [44], Belgium [32], and Barcelona [45], the US [42], and Japan [18]. This could be related to the increased rates of MSAF and birth asphyxia following induction of labor.

Our study showed that the rate of NICU admission among babies born through induction of labor (15.4%) was significantly higher compared to spontaneously born newborns (5.7%). This result is similar to the studies conducted in Jordan [46], Australia [9, 29], India [16, 31], Switzerland [28], Belgium [32], and Nigeria [39, 40]. This finding could be due to the fact that induction of labor results in early neonatal complications that need special care in the NICU [10, 17, 30, 38].

In this study, mothers who had no formal education were two times more likely to develop adverse neonatal outcomes compared to women who had studied higher than secondary school. This finding is comparable to the studies conducted in India [19], Italy [20], the USA [22], and Ethiopia [21]. This association might be due to the fact that the level of maternal education has been identified as being important for making prompt decisions during complications [19]. During either obstetrics or other health care counseling sessions, mothers with higher grades comprehend the information better than uneducated mothers [47].

The odds of adverse neonatal outcomes among participants who have had a chronic disease were nearly four times higher compared to those who have not had a chronic medical disease. This finding is comparable to the studies conducted in Denmark [48] and Ethiopia [49]. This might be associated with the fact that chronic diseases diminish feto-placental placental blood supply [15].

When compared to those whose male partner was involved in their health-seeking decision, the likelihood of adverse neonatal outcomes was nearly two-fold higher among participants whose male partner was not involved. This finding is congruent with the study findings from Australia [50] and Kenya [51]. The male partners’ involvement in women’s healthcare is a key to emotional, physical, and financial support for childbearing women [52].

In this study, gestational age was also shown to have a significant association with adverse neonatal outcomes. The odds of developing adverse neonatal outcomes were nearly ten-fold higher among participants who experienced preterm labor compared to those who experienced term labor. This finding is comparable to a study conducted in Israel [53]. The reason might be due to the immaturity of the newborn, such as fetal lung immaturity [54]. Similarly, the odds of adverse outcomes among newborns delivered in the post-term period were around two-fold higher than those born in the term period. This result is comparable to a study conducted in Addis Ababa [55]. This association might be due to the fact that there is a decline in the utero-placental blood supply in post-term pregnancy [56].

The current study revealed a significant association between adverse neonatal outcomes and mode of delivery (operative deliveries). Accordingly, the odds of developing adverse neonatal outcomes were nearly nine fold higher among participants who gave birth through instrumental delivery compared to those who gave birth through SVD. Likewise, participants who gave birth through CS were about four times more likely to experience adverse neonatal outcomes compared to those who gave birth through SVD. This association is similar to a study conducted in Sekota [49]. The reason for these associations might be related to the fact that operative deliveries were conducted, in particular, due to complications of the fetus or/and the mother [9].

The likelihood of adverse neonatal outcomes was about five times higher among participants who developed labor-delivery complications compared to their counterparts. This finding is comparable to a study conducted in Sweden [54]. This significant association could be due to the effects of both the labor process and the iatrogenic management of labor complications [54].

## Limitations

The current study’s utilization of a large sample size, which ensures its external validity, is strength. However, the study’s shortcoming is that it is cross-sectional in nature. The possible adverse neonatal outcomes that could occur after 24 h of birth were not taken into account in this study. Hence, this might underestimate the proportion of adverse neonatal outcomes.

## Conclusion

In the study area, one in five newborns develops adverse neonatal outcomes within 24 h of birth. The adverse neonatal outcomes among women who gave birth through induced labor were significantly higher compared to those of women who gave birth through spontaneous labor. In addition, immediate newborn death and fetal death during labor, NICU admission, the need for resuscitation, the first minute and fifth minute Apgar scores less than 7, and neonatal jaundice more frequently occurred among women who gave birth through induced labor than in women who gave birth through spontaneous labor. Being uneducated, non-involvement of the male partners, the presence of chronic disease, preterm and post-term delivery, complications during labor, and operative delivery (instrumental and CS) were found to be significant factors enhancing adverse neonatal outcomes.

### Implication for practice

Any iatrogenic intervention in labor has its own unfavorable effects on the mother and the newborn in comparison to a natural termination. One of the iatrogenic obstetric methods is labor induction. We found that, compared to spontaneous labor, induced labor had a considerably higher proportion of unfavorable newborn outcomes.

In addition, the study identified the factors determining adverse neonatal outcomes. The labor-management and newborn care teams must therefore anticipate potentially adverse neonatal outcomes with caution and be alertly prepared to handle them if induction of labor is indicated and inevitable. In order to prevent adverse consequences early on, it is also essential to spot risky cases during both the prenatal and intrapartum periods.

## Data Availability

The data and materials used in this study are available from the corresponding authors upon reasonable request.

## Abbreviations

ANC: Antenatal Care
APGAR: Appearance, Pulse Rate, Grimace, Activity & Respiratory Rate
CS: Cesarean Section
IOL: Induction of Labor
IUFD: Intrauterine Fetal Death
MUAC: Mid Upper Arm circumference
NICU: Neonatal Intensive Care Unit
NRFHRP: Non-Reassuring Fetal Heart Rate Pattern
US: United States
